# Brain metastases from colorectal cancer – a retrospective dual center study

**DOI:** 10.1007/s11060-026-05536-0

**Published:** 2026-03-24

**Authors:** S. Müller, A. Hendricks, K. Uttinger, M. Kostatin, M. Brüggemann, M. Schrader, B. Polat, S. Flemming, J. F. Lock, C.-T. Germer, A. Wiegering, U. Pession

**Affiliations:** 1 Department of General, Visceral, Transplant, Vascular and Paediatric Surgery, University Hospital Wuerzburg, 97080 Wuerzburg, Germany; 2 Comprehensive Cancer Center Mainfranken, University of Wuerzburg, 97080 Wuerzburg, Germany; 3 Department of General, Visceral, Transplant and Thoracic Surgery, Goethe University Frankfurt, 60596 Frankfurt, Germany; 4 Department of Radiation Oncology, University Hospital Wuerzburg, 97080 Wuerzburg, Germany

**Keywords:** CRC, Colorectal cancer, Brain metastases, Tumour

## Abstract

**Background:**

Although brain metastases (BM) represent an uncommon manifestation of colorectal cancer (CRC), their occurrence is associated with a markedly reduced life expectancy. Advances in systemic therapies and neuroimaging have led to a growing number of detected cases. However, data on prognostic markers and optimal management still remain limited. This dual center analysis from two major German cancer centers sought to describe clinical characteristics, treatment approaches and survival determinants in patients with CRC who developed BM.

**Methods:**

All individuals diagnosed with CRC and subsequent BM between January 2000 and December 2024 were retrospectively identified from the tumor registries of the University Hospitals Wuerzburg and Frankfurt. Demographic, pathological and therapeutic parameters were extracted and analyzed. Overall survival following BM diagnosis was assessed using a multivariable Cox proportional hazards model with backward stepwise likelihood ratio selection (entry criterion *p* < 0.05, removal *p* > 0.10). Hazard ratios (HR) and 95% confidence intervals (CI) were calculated.

**Results:**

The study included 279 patients (Wuerzburg 48.4%, Frankfurt 51.6%). The mean age at CRC diagnosis was 62.1 years and at BM diagnosis 65.1 years; 61.6% of the patients were male. The median interval between CRC diagnosis and BM detection in metachronous patients was 40.5 months, with 11.1% exhibiting synchronous BM. Liver, lung, and bone metastases were present in 50.9%, 64.2%, and 22.6% of patients, respectively. Among tested cases, KRAS mutations were found in 54.2%. Surgical resection of BM was undertaken in 36.2%, and radiotherapy in 67.0%. On multivariable analysis, Karnfosky Performance Status emerged as independent predictors of prolonged survival (HR = 0.98; 95% CI 0.97-1-00; *p* = 0.008). Brain metastasis surgery showed a borderline association with improved survival (HR 0.51; 95% CI 0.26–1.00; *p* = 0.050). Median survival from the time of BM diagnosis was 20 months (95% CI 14.140–25.860) in the operated group compared with 3 months (95% CI 2.089–3.911) in the non-operated group.

**Conclusion:**

Patients with CRC who develop BM are a subgroup with heterogeneous courses of disease. In addition to KPS, surgical resection of BM was associated with improved survival. This emphasizing the benefit of local treatment in appropriately cases. Collaborative, prospective studies are required to validate these findings and to refine therapeutic strategies for this rare entity.

## Introduction

Colorectal cancer (CRC) ranks as the third most frequently diagnosed malignancy worldwide and the second most common in Germany, accounting for approximately 1.9 million new cases and 930,000 deaths worldwide in 2020 [1]. At initial diagnosis, around one-quarter of patients present with distant metastases, an additional 25% develop metastatic disease later on [2]. Predominant sites for metastasis are liver and lung, whereas brain metastases (BM) occur in 1–4% of patients. Nevertheless, their incidence appears to be increasing, likely reflecting prolonged survival due to advances in systemic therapy and enhanced detection through modern neuroimaging [3–6].

The development of BM typically indicates advanced systemic disease and is associated with poor prognosis, with a median survival time rarely exceeding one year [3, 6]. Neurological symptoms — such as headaches, seizures, or focal deficits — frequently impair quality of life and complicate both treatment and supportive care. Current management strategies include surgical resection, stereotactic radiosurgery, and whole-brain radiotherapy, often complemented by systemic approaches such as targeted agents or immunotherapy [1, 3, 5].

Given the rarity of CRC-related BM and the scarcity of robust evidence, optimal therapeutic strategies remain uncertain. There is a pressing need to identify prognostic and predictive factors, to refine patient selection for aggressive local therapies and to develop systemic regimens capable of penetrating the blood–brain barrier. In the present study, we retrospectively analyzed clinical characteristics and treatment outcomes of CRC patients with BM treated at two high-volume cancer centers.

## Methods

### Data source

Patients with colorectal carcinoma who had been diagnosed with BM between January 1, 2000, and December 31, 2024, were identified through the tumor registry of the Comprehensive Cancer Center Mainfranken. Corresponding patient characteristics were obtained from the database of the University Hospital Frankfurt, with the difference that the initial diagnosis of CRC began there on January 1, 2004. Ethical approval was granted by the respective institutional review boards (Wuerzburg: [20210118 01]; Frankfurt: [2025-2240-iBDF 274/18]). All data were transferred into an Excel database and pseudonymized prior to analysis.

### Baseline comparisons

Baseline characteristics were compared between centers. Categorical variables (sex, KRAS mutation status, presence of liver, lung, or bone metastases, brain metastasis surgery, radiation therapy, onset of BM and survival status) were analyzed using the χ² test, and results are presented as counts and percentages. Continuous variables (age at diagnosis of CRC and of BM, time to BM) were compared between centers using independent samples t tests. Effect sizes and 95% confidence intervals (CIs) were calculated.

KRAS analyses were performed using tissue from the primary tumour. No data were available on whether the mutation was also present in the BM.

Patient-related data were obtained from the clinical documentation systems and the tumour registry. As data collection began in 2000, patients for whom no date of death was available were not contacted. Considering the advanced stage of disease and the likelihood that many patients had already died, contacting patients could have caused distress to relatives and was therefore not pursued.

### Survival analyses

Overall survival after diagnosis of BM was analyzed using a Cox proportional hazards regression model. Survival time was defined as time from diagnosis of BM to death; patients alive at last follow-up were censored. Candidate covariates included sex, age at diagnosis of BM, synchronous versus metachronous presentation, KRAS status, brain metastasis surgery, number of BM, Karnofsky Performance Status (KPS), number of metastatic sites, and radiotherapy modality. Continuous variables were analyzed on their original scale and categorical variables were modelled using indicator coding. Model selection was performed using a stepwise likelihood ratio method (entry criterion *p* < 0.05, removal criterion *p* > 0.10). Hazard ratios (HRs) with 95% CIs were reported. Survival and hazard functions were graphically displayed. Results from both the full multivariable model including all candidate variables and the final model obtained by backward stepwise selection are reported. This approach allows presentation of the fully adjusted analysis while also providing a parsimonious model highlighting the variables independently associated with survival.

All statistical analyses were performed using IBM SPSS Statistics (version 29, IBM Corp., Armonk, NY, USA).

## Results

A total of 279 patients with colorectal cancer (CRC) and subsequent BM were included in the analysis, with 135 (48.4%) treated in Wuerzburg and 144 (51.6%) in Frankfurt. In Wuerzburg, the prevalence of BM among patients with colorectal carcinoma was 1.91%, whereas in Frankfurt the prevalence could not be determined accurately due to missing data. The mean age at CRC diagnosis was 62.1 years and the mean age at BM diagnosis was 65.1 years, with comparable age distributions between centers. Overall, 61.6% of patients were male and 58.4% were female. Synchronous BM were present in 11.1% of patients, while in cases with metachronous BM the mean interval between CRC and BM diagnosis was 40.5 months.

Across most clinical, pathological, and treatment-related parameters, no significant differences were observed between the two centers.

Rectal cancer was the primary tumour site in 48.4% of cases. Systemic metastases were common, with liver metastases observed in 50.9%, lung metastases in 64.2%, and bone metastases in 22.6% of patients. Molecular profiling was available in 41.9% of cases, revealing KRAS mutations in 54.2% and wild-type KRAS status in 45.8%.

**Fig. 1:**
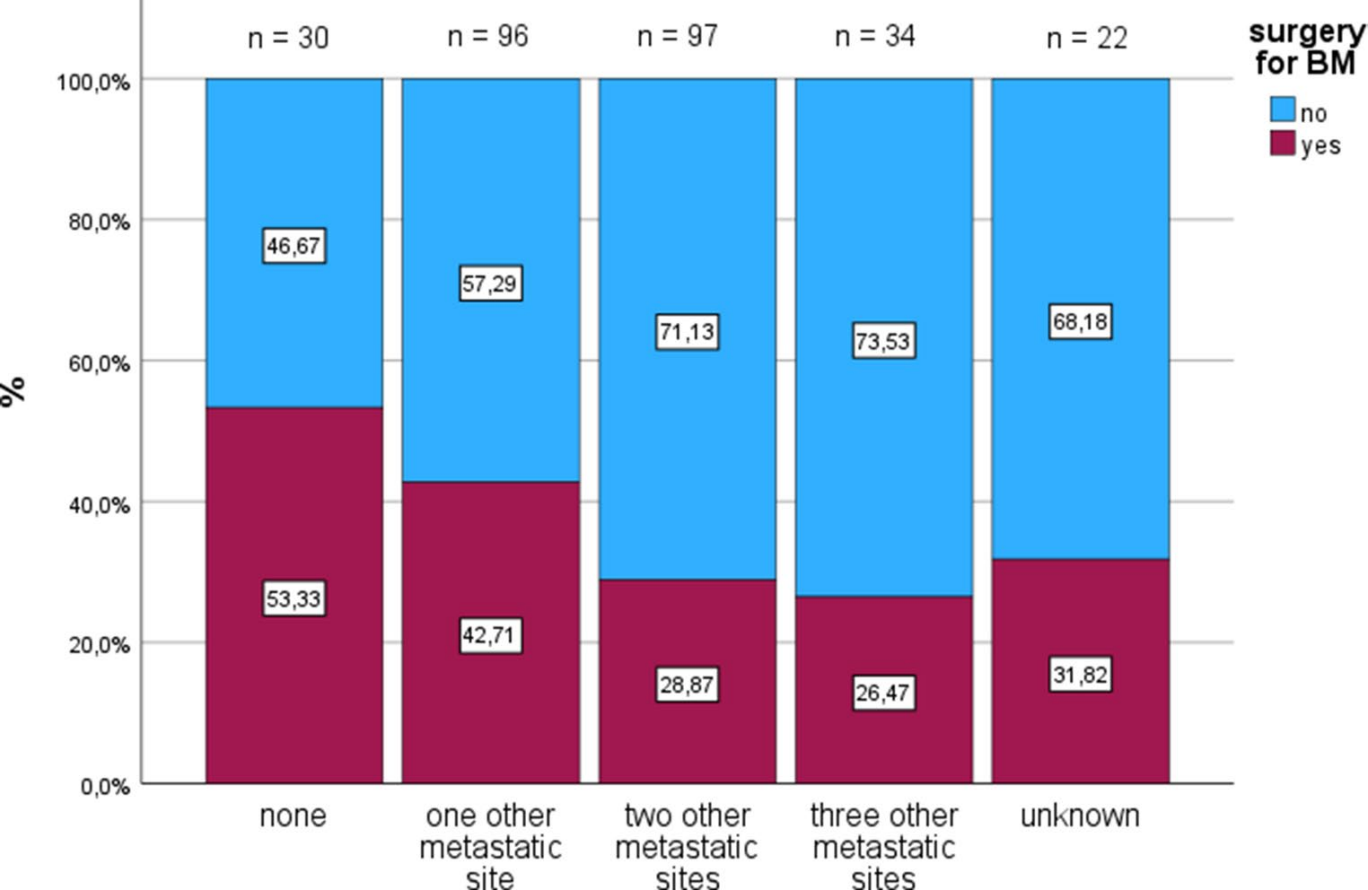
Distribution of metastatic sites between the surgery and non-surgery groups

Surgical resection of BM was performed in 36.2% of patients. Patients undergoing surgery more often had a single BM, whereas multiple metastases were more common without surgery (*p* < 0.001). Overall, 67.0% of patients received radiotherapy, with no significant difference between Wuerzburg and Frankfurt (*p* = 0.316). Primary radiotherapy was more frequent in Wuerzburg, whereas postoperative radiotherapy was more common in Frankfurt. The distribution of radiation techniques differed significantly between centers (*p* = 0.018), with higher use of WBRT and WBRT with boost in Wuerzburg. Patients who did not undergo surgical resection more frequently presented with liver metastases (58.4% vs. 37.6%, *p* < 0.001) and bone metastases (26.4% vs. 15.8%, *p* = 0.036), whereas the prevalence of lung metastases was similar between groups (*p* = 0.945). This finding is also illustrated in Fig. 1. The distribution of additional extracranial metastatic sites differed significantly between patients with and without surgical treatment (*p* = 0.015). No significant differences were observed between treatment groups (with or without surgery) regarding age at CRC or BM diagnosis, sex distribution, time to BM development, synchronous BM presentation, or primary tumour location.

Baseline characteristics are summarized in Tables 1 and 2.

**Fig. 2:**
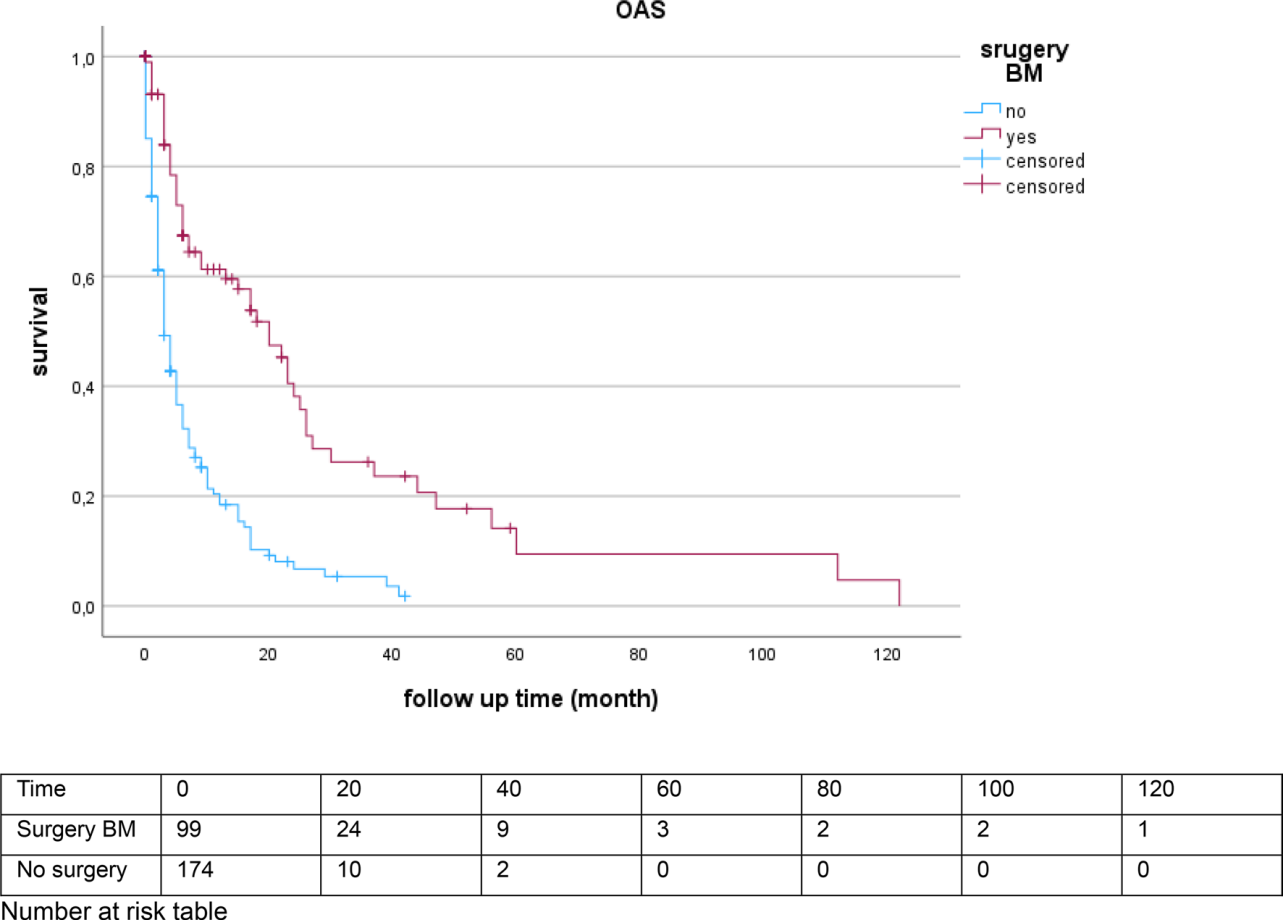
Kaplan–Meier survival curves comparing overall survival (OAS) for patient with BM Surgery [20 months (95% CI 14.140–25.860)] and no surgery [3 months (95% CI 2.089–3.911)]

The univariate Kaplan–Meier analysis showed a median overall survival of from the time of BM diagnosis was 20 months (95% CI 14.140–25.860) in the operated group compared with 3 months (95% CI 2.089–3.911) in the non-operated group (Fig. 2).

A multivariate Cox proportional hazards regression model was performed to identify independent prognostic factors for overall survival after the diagnosis of BM. Variables entered into the model included, sex, age at BM, synchronicity of metastasis (synchronous vs. metachronous), *KRAS* mutation status, surgical resection of BM, number of BM and modality of radiotherapy, number of extracranial metastatic sites and KPS.

Of 279 patients in the source cohort, 109 (39.1%) had complete data and were included in the multivariable Cox regression analysis. Among these, 84 patients experienced the event of interest and 25 were censored.

In the final multivariable Cox model (Table 3), higher KPS was significantly associated with improved survival (HR 0.98 per point increase, 95% CI 0.97–1.00; *p* = 0.008). Surgical resection of BM showed a borderline association with improved survival (HR 0.51; 95% CI 0.26–1.00; *p* = 0.050). No significant associations were observed for sex, age at diagnosis of BM, synchronous versus metachronous presentation, KRAS status, the number of BM, or the number of extracranial metastatic sites. Radiotherapy modality was not significantly associated with survival in the multivariable model (global *p* = 0.390). None of the individual radiotherapy indicator terms showed a consistent statistically significant association. To further explore a parsimonious model, backward stepwise selection was performed. In the final multivariable model (Table 4), both surgical resection of BM (HR 0.60; 95% CI 0.36–0.98; *p* = 0.043) and KPS (HR 0.98 per point increase, 95% CI 0.97–1.00; *p* = 0.021) remained independently associated with improved survival.

## Discussion

In this retrospective analysis of 279 patients with colorectal cancer (CRC) and subsequent BM treated at two high-volume German cancer centers, we confirm several important observations and raise clinically relevant points that merit further investigation. Overall, our findings reinforce that BM in CRC represent a rare but clinically significant event, with survival largely determined by performance status and the feasibility of local therapeutic interventions, particularly surgical resection.

### Epidemiology and incidence patterns

Consistent with prior reports, the incidence of BM in CRC is low (typically 1–4%), though there is some evidence that rates may be increasing in the era of prolonged systemic therapy and more sensitive neuroimaging [3, 6, 7]. This is important because improved systemic control of extracranial disease may allow intracranial disease to become more clinically evident.

In our cohort, the prevalence of BM differed substantially between centers. In Wuerzburg, BM were observed in 1.91% of CRC patients between 2000 and 2024 (133/7553), which is in line with population-based estimates. In contrast, the incidence in Frankfurt was 5.55% (144/2618) for the period 2004–2024. This discrepancy is most plausibly explained by referral bias: while CRC is treated across multiple regional hospitals, neurosurgical management of BM is centralized at the university hospital in Frankfurt, leading to an overrepresentation of BM cases. Conversely, Wuerzburg functions as a primary regional provider, likely yielding a more accurate reflection of true population-level incidence.

The temporal pattern of BM development further supports their role as a late manifestation of systemic disease. In our cohort, synchronous BM occurred in approximately 11.1% of patients, while metachronous BM developed after a mean interval of 40.5 months. BM were frequently accompanied by extracranial metastases, particularly in the lung (64.2%), liver (50.9%), and bone (22.6%), consistent with established metastatic pathways [2].

Notably, patients in our cohort were younger at CRC diagnosis than expected based on German population-based data from the Robert Koch Institute (median age 71 years in men and 75 years in women). This likely reflects selection effects inherent to tertiary care centers, where younger and fitter patients are preferentially referred, while older patients may receive treatment in non-specialized institutions [8].

### Prognosis and impact of local therapy

The prognosis of patients with CRC-associated BM remains poor. Previous studies report median overall survival ranging from 2 to 10 months, with only a minority of patients surviving beyond one year [3, 6]. Our findings are consistent with these observations but further highlight the prognostic relevance of local treatment strategies.

In multivariable analysis, surgical resection of BM was associated with improved survival. Patients undergoing surgery achieved a median survival of 20 months, compared to only 3 months in non-surgically treated patients. While this association must be interpreted in the context of selection bias, it aligns with existing evidence suggesting that carefully selected patients benefit from aggressive local treatment approaches, including surgery and stereotactic radiotherapy [9].

These findings are further supported by prospective and registry-based data. The METACER cohort study reported a median overall survival of 7.1 months in CRC patients with BM, identifying performance status and surgical intervention as key prognostic factors [10]. Similarly, data from the Vienna Brain Metastasis Registry indicate median survival times of 5–8 months, with improved outcomes in patients receiving local therapies and those with preserved functional status [11].

From a clinical perspective, two distinct patterns of disease progression can be observed. In one subgroup, BM occur in the context of widespread systemic disease, and survival is primarily limited by extracranial progression. In another subgroup, BM develop despite limited or controlled extracranial disease, with neurological deterioration becoming the dominant cause of death. Importantly, BM are generally less responsive to systemic chemotherapy compared to hepatic or pulmonary metastases, underscoring the critical role of local therapies in this setting.

### Implications for clinical management

Our findings have several implications for clinical practice. First, given the overall low incidence of BM, routine brain imaging in unselected CRC patients is not justified. However, in patients with high-risk features—such as rectal primaries, lung metastases, or KRAS mutations—earlier neuroimaging may be warranted to facilitate timely detection [6, 12].

Second, surgical resection should be strongly considered in selected patients with favorable prognostic characteristics, including good performance status, limited extracranial disease, and solitary or oligometastatic brain involvement. These decisions require careful multidisciplinary evaluation within specialized neuro-oncological teams.

Third, there remains a substantial unmet need for effective systemic therapies with intracranial activity. Although factors such as KRAS mutation status, radiotherapy, and timing of BM (synchronous vs. metachronous) were not independent prognostic variables in our analysis, emerging molecular stratification and novel therapeutic agents may influence outcomes in the future.

### Methodological considerations and center-specific differences

Several differences between the participating centers warrant consideration. In Wuerzburg, detailed radiotherapy data could be retrieved, whereas documentation in Frankfurt was limited in some cases to the presence or absence of radiotherapy without specification of modality. Furthermore, follow-up completeness differed substantially, with known dates of death available for 85% of patients in Wuerzburg compared to only 41% in Frankfurt, where a significant proportion of patients were lost to follow-up. These discrepancies may have influenced survival estimates and should be considered when interpreting the results.

Another limitation concerns the level of detail available for surgical and radiotherapy data. While it was recorded whether patients underwent surgical resection, information on R0 status, the urgency of surgery, or whether all known BM were removed was not available. Radiotherapy was categorized by modality, but detailed data on dose, fractionation, and treatment intent were not available for enough patients to be included in the analyses.

### Limitations

Our study has a number of limitations that must be acknowledged. The retrospective design introduces potential for selection bias (e.g., only patients who underwent surgery survived long enough to be selected). The completeness of data varied between centers (e.g., survival status known in 85.9% in Wuerzburg vs. 41.0% in Frankfurt). This differential data completeness may influence our findings. In addition, detailed information on performance status (e.g., KPS or ECOG), number and size of brain lesions, extracranial disease burden at the time of BM diagnosis, and subsequent systemic therapies were not uniformly available, factors which are known to influence prognosis. Of 279 patients, only 109 (39.1%) had complete data and were included in the multivariable Cox analysis (84 events, 25 censored). Inclusion of additional variables would have further reduced the sample size due to missing data; therefore, the model was restricted to preserve statistical power. Consequently, surgery was analyzed as a binary variable without incorporating details on number of resected lesions, resection status, or urgency, and radiotherapy was not further specified by dose or fractionation.

While the association between surgical resection of BM and improved overall survival should be interpreted with caution due to inherent selection bias, the finding remains clinically meaningful. Patients selected for surgery generally exhibited favorable prognostic characteristics, which likely contributed to improved outcomes. Nevertheless, the consistent survival benefit observed across analyses suggests that, within a carefully selected patient population, surgical resection represents an important therapeutic option. Finally, the generalizability of our results may be limited to tertiary cancer center populations with access to neurosurgical and radio surgical services.

Data collection began in 2000, at a time when patients were not yet treated with the multimodal approaches that are standard today. In addition, BRAF and MSI testing were not incorporated into the German S3 guidelines until 2014, while HER2 testing was not recommended until 2021. Consequently, molecular markers that are routinely assessed in current clinical practice could not be evaluated adequately in the present study.

### Future directions

Going forward, prospective multicenter registries or consortium efforts are needed to better characterize CRC-BM. Specifically, research should focus on: (i) refining prognostic and predictive biomarkers (including molecular subtypes, *KRAS/NRAS/BRAF*, Her2neu [13], microsatellite status) in this population; (ii) assessing the utility and cost-effectiveness of routine brain imaging in high-risk CRC subsets; (iii) exploring systemic therapies (targeted, immunotherapy, antibody-drug conjugates) with intracranial activity; and (iv) developing consensus guidelines for the multidisciplinary management of CRC-BM, including criteria for surgical and radio surgical intervention [4, 5, 14].

## Conclusion

In conclusion, our study reinforces the notion that BM in CRC, although uncommon, represents a clinically important entity. Our finding that surgical resection of BM is associated with improved survival underscores the need for early recognition and aggressive multidisciplinary management in selected patients. Continued research is warranted to improve outcomes for this vulnerable patient group.

**Table 1:** Baseline characteristics between centers

	All *n* = 279	Wuerzburg *n* = 135	Frankfurt *n* = 144	*p*
Age CRC years				
Mean (SD)	62.06 (10.25)	62.52 (10.45)	61.63 (10.07)	0.987
Age BM years				
Mean (SD)	65.05 (10.22)	65.18 (10.33)	64.92 (10.16)	0.992
Male Female	173 (61.6) 106 (38.4)	80 (59.3) 55 (40.7)	93 (64.6) 51 (35.4)	0.360
Mean time to BM month (SD)	35.68 (30.57)	31.81 (27.73)	39.32 (32.69)	0.040
Synchronous	31 (11.1)	16 (11.9)	15 (10.4)	0.448
KRAS				
Mutation Wildtype Not tested	60 (21.5) 57 (20.4) 162 (58.1)	39 (28.9) 33 (24.4) 63 (46.7)	21 (14.6) 24 (16.7) 99 (68.7)	0.430
Lung metastases	179 (64.2)	88 (65.2)	91 (63.2)	0.084
Liver metastases	142 (50.9)	67 (49.6)	75 (50.1)	0.417
Bone metastases	63 (22.6)	35 (25.9)	28 (19.4)	0.067
Surgery for BM	101 (36.2)	41 (30.4)	60 (41.7)	0.050
Radiation Primary Postoperative Unknown	187(67.0) 85 (29.4) 55 (45.5) 47 (25.1)	95 (70.4) 63 (66.3) 31 (32.6) 2 (2.1)	92 (63.9) 22 (23.9) 24 (26.1) 45 (48.9)	0.316
STx WBRT WBRT + boost PBRT Unknown	50 (26.7) 50 (26.7) 28 (15.0) 16 (8.6) 43 (23.0)	25 (26.3) 32 (33.7) 23 (24.2) 11 (11.6) 4 (4.2)	25 (27.2) 18 (19.6) 5 (5.4) 5 (5.4) 39 (42.4)	0.018
Documented date of death	175 (62.7)	116 (85.9)	59 (41.0)	< 0.001
Rectal cancer	135 (48.4)	62 (45.9)	73 (50.7)	0.426
Location of CRC				
Right Colon Transverse colon Left Colon Rectum Unknown	42 16 56 145 15	9 12 29 68 12	33 4 27 77 3	

Values are n (%) unless otherwise indicated. SD = Standard Deviation, BM = brain metastases, STX = stereotactic radiotherapy, WBRT = whole brain radiotherapy, PBRT = partial brain radiotherapy

**Table 2:** Baseline Characteristics surgery vs. no surgery

	All *n* = 279	Surgery for BM *n* = 101	No surgery *n* = 178	*p*
Lung metastases	179 (64.2)	66 (65.3)	113 (63.5)	0.945
Liver metastases	142 (50.9)	38 (37.6)	104 (58.4)	< 0.001
Bone metastases	63 (22.6)	16 (15.8)	47 (26.4)	0.036
Extracranial metastases				
No other metastases One other metastatic site Two other metastatic sites Three other metastatic sites Unknown other metastases	30 (10.8) 96 (34.4) 97 (34.8) 34 (12.2) 22 (7.9)	16 (15.8) 41 (40.6) 28 (27.7) 9 (8.9) 7 (6.9)	14 (7.9) 55 (30.9) 69 (38.8) 25 (14.0) 15 (8.4)	0.52
Age CRC (SD) years	62.06 (10.25)	61.18 (9.23)	62.56 (10.77)	0.281
Age BM (SD) years	65.05 (10.22)	63.84 (9.49)	65.73 (10.58)	0.138
Male Female	173 (61.6) 106 (38.4)	63 (62.4) 38 (37.6)	110 (61.8) 68 (38.2)	0.924
KPS points (SD)	72.05 (21.91)	78.69 (20.93)	67.94 (21.55)	< 0.001
Mean time to BM month (SD)	35.68 (30.57)	32.12 (25.59)	37.71 (32.96)	
Synchronous	31 (11.1)	12 (11.9)	19 (10.7)	0.758
Number of BM				
One Two Multiple	153 18 82	79 8 10	74 10 72	< 0.001
Rectal Cancer	135 (48.4)	43 (42.6)	92 (51.7)	0.143

Values are n (%) unless otherwise indicated. SD= Standard Deviation. BM = brain metastases, CRC = colorectal Cancer, KPS = Karnofsky Performance Status

**Table 3:** Variables Cox regression

Variable	B (SE)	Wald χ²	HR (Exp(B))	95% CI for HR	*p*-value
Sex (male vs. female)	-0.001 (0.240)	0.000	1.00	0.62–1.60	0.997
Age at BM (years)	-0.001 (0.013)	0.005	1.00	0.97–1.03	0.941
Synchronous vs. metachronous BM	0.502 (0.381)	1.732	1.65	0.78–3.49	0.188
KRAS mutation (yes vs. wild type)	-0.140 (0.238)	0.348	0.87	0.55–1.39	0.555
Surgical resection of BM (yes vs. no)	-0.684 (0.349)	3.837	0.51	0.26–1.00	0.050
Number of BM	-0.031 (0.138)	0.050	0.97	0.74–1.27	0.823
KPS	-0.020 (0.007)	6.975	0.98	0.97–1.00	0.008
Number of extracranial metastatic sites	-0.214 (0.168)	1.628	0.81	0.58–1.12	0.202
Radiotherapy modality (overall)	-	6.302	-	-	0.390

Reference categories were: female sex, metachronous brain metastases, KRAS wild type, no surgical resection of brain metastases, Hazard ratios < 1 indicate reduced risk of death compared with the reference category. Radiotherapy modality was entered as a categorical variable using indicator coding; the p-value represents the global Wald test for the variable

BM = brain metastases, KPS = Karnofsky Performance Status, B (SE) = Regression coefficient (standard error), HR (Exp(B)) = Hazard Ratio (exponentiated regression coefficient), *95%CI*, 95% confidence interval,

**Table 4:** Final multivariable Cox model

Variable	B (SE)	Wald χ²	HR (Exp(B))	95% CI for HR	*p*-value
Surgical resection of BM (yes vs. no)	-0.520 (0.257)	4.102	0.60	0.36–0.98	0.043
KPS	-0.016 (0.007)	5.351	0.98	0.97–1.00	0.021

BM = brain metastases, KPS = Karnofsky Performance Status, B (SE) = Regression coefficient (standard error), HR (Exp(B)) = Hazard Ratio (exponentiated regression coefficient), *95%CI*, 95% confidence interval,

## Data Availability

No datasets were generated or analysed during the current study.
